# Supplementary material for: Diagnostic Aqueous Humor Proteome Predicts Metastatic Potential in Uveal Melanoma
Source: Int J Mol Sci. 2023 Apr 6;24(7):6825. doi: 10.3390/ijms24076825 (PMC10094875; doi:10.3390/ijms24076825)
Supplement: Supplementary file 1 [file ijms-24-06825-s001.zip › Supplementary Figures.pdf]

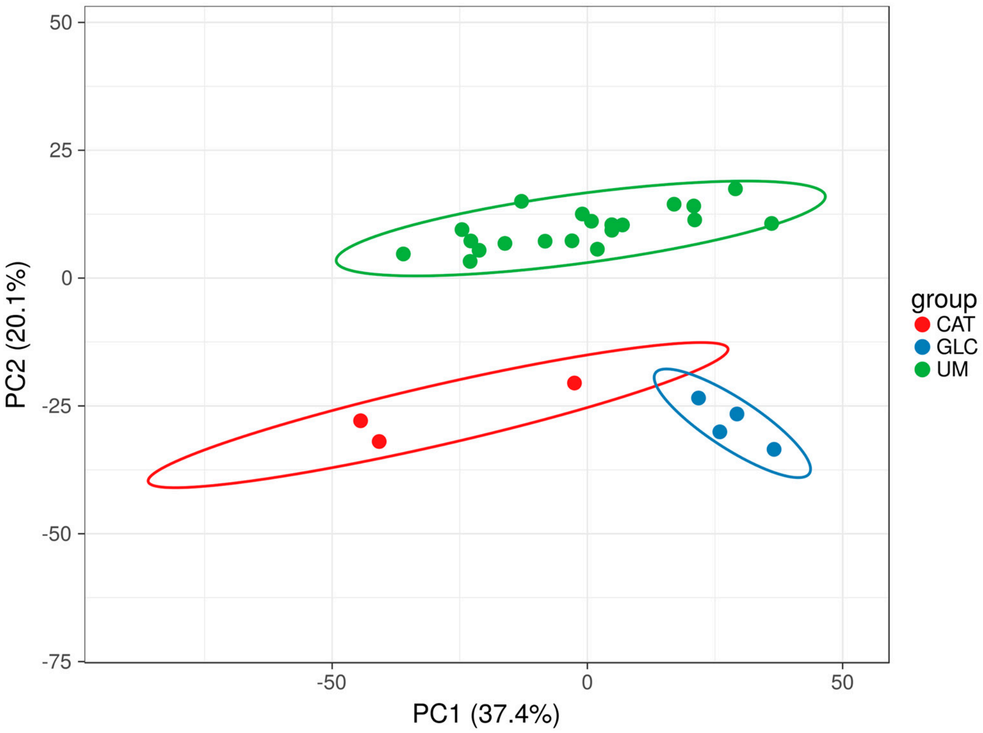

Figure S1. Principal component analysis of 1469 normalized protein expression (NPX) distribution from 3 congenital cataract (CAT), 4 congenital glaucoma (GLC) and 20 uveal melanoma (UM) aqueous humor (AH) samples. Three distinct clusters by disease types were indicated.

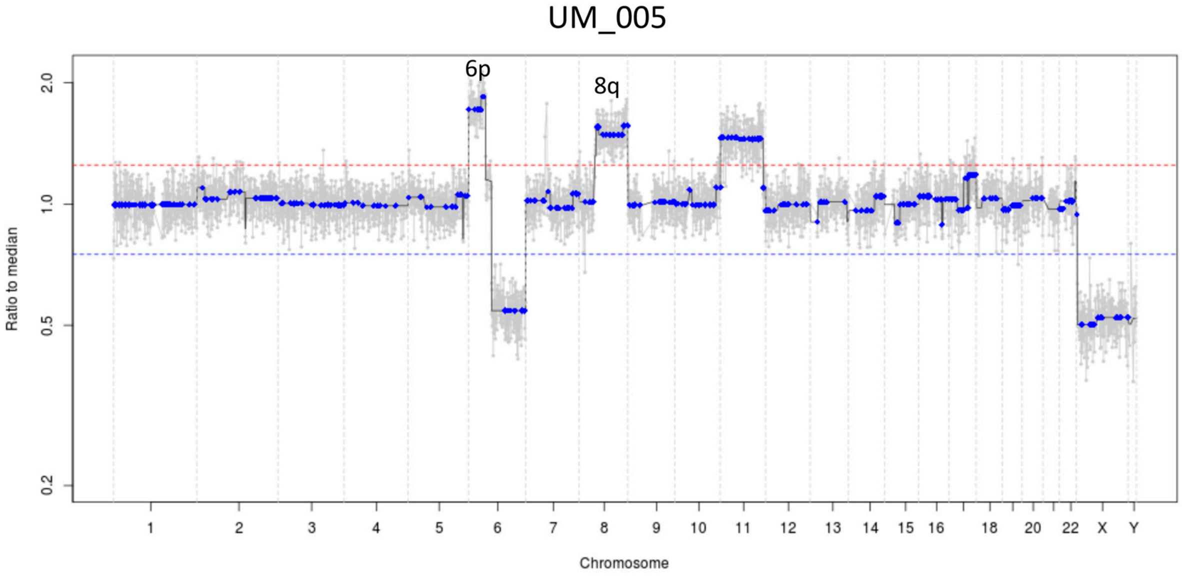

Figure S2. Somatic copy number alterations (SCNAs) in patient uveal melanoma (UM_005) aqueous humor (AH) samples. The ratio to median equals to "1" denotes two-copies of chromosomes. Gains and losses of chromosomes were shown. Highly recurrent UM SCNAs of 6p gain and 8q gain were highlighted. Red-dashed and blue-dashed lines represent 20% deflection from a baseline human genome. The ratio to median above red line or below blue line were considered SCNA positive.

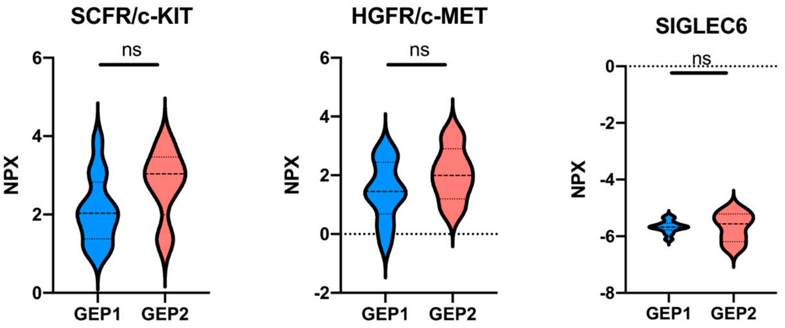

Figure S3. The normalized protein expression (NPX) levels of the 3 prognostic biomarkers identified in vitreous humor (VH) from Velez et al. Protein levels of SCFR, HGFR and SIGLEC6 were compared between gene expression profile (GEP) classes. Mann-Whiney U-test was used for data comparison. ns, non-significant. Gene expression profile 1 (GEP1): low likelihood of metastasis; gene expression profile 2 (GEP2): high likelihood of metastasis; gene expression profile unknown (GEP NA): limited access of tumor biopsy for performing GEP analysis.

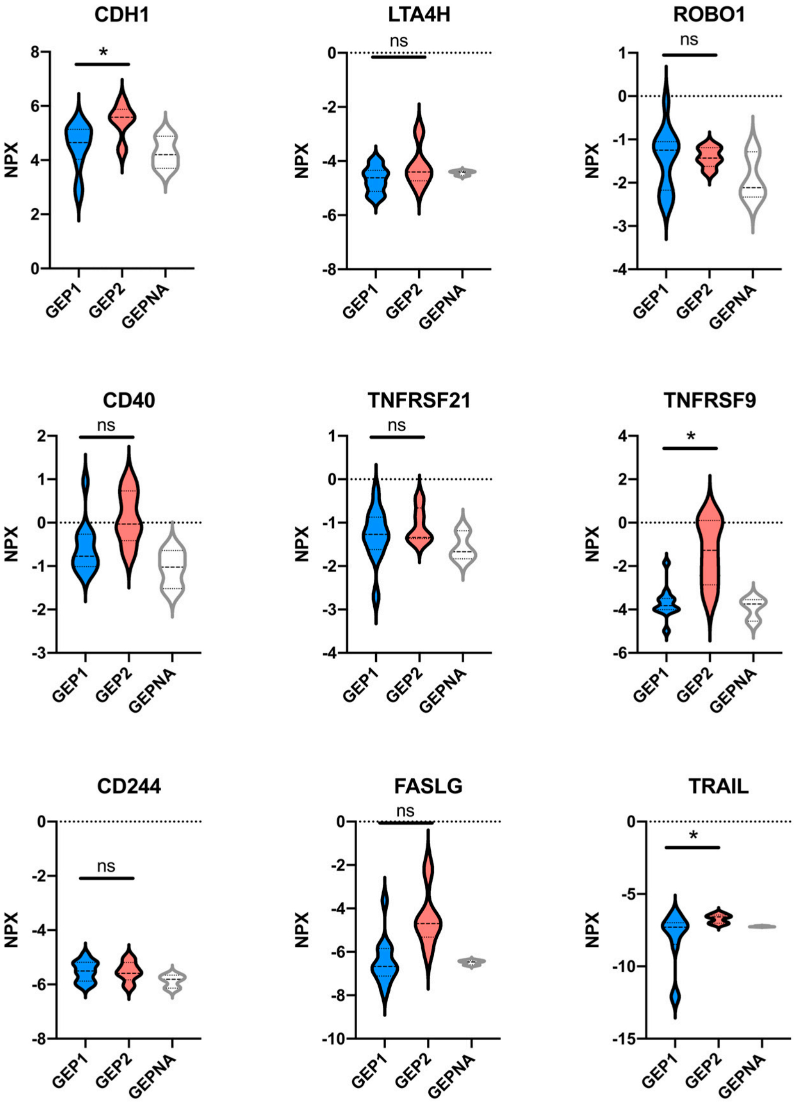

Figure S4. The normalized protein expression (NPX) protein expression levels of the 3 gene expression profile (GEP) signature genes and the 6 prognostic biomarkers identified in aqueous humor (AH) from Wierenga et al. Protein levels of the 3 GEP signature genes: CDH1, LTA4H and ROBO1, and the 6 prognostic biomarkers: CD40, TNFRSF21, TNFRSF9, FASLG, CD244 and TRAIL were compared between GEP classes. Mann-Whiney U-test was used for data comparison. ns, non-significant; *, p values < 0.05. Gene expression profile 1 (GEP1):

low likelihood of metastasis; gene expression profile 2 (GEP2): high likelihood of metastasis; gene expression profile unknown (GEP NA): limited access of tumor biopsy for performing GEP analysis.
